# New Polymerizable Tetraaza Macrocycle Containing Two Acridine Units for Selective Fluorescence Sensing of Metal Ions

**DOI:** 10.1007/s10895-021-02851-9

**Published:** 2021-12-29

**Authors:** Ádám Golcs, Korinna Kovács, Panna Vezse, Péter Huszthy, Tünde Tóth

**Affiliations:** 1 Department of Organic Chemistry and Technology, Budapest University of Technology and Economics, Szent Gellért tér 4, 1111 Budapest, Hungary; 2 Institute for Energy Security and Environmental Safety, Centre for Energy Research, Konkoly-Thege Miklós út 29-33, 1121 Budapest, Hungary

**Keywords:** Acridine, Metal ion recognition, Fluorescence sensor, Aza macrocycles

## Abstract

A new fluorescent bis(acridino)-macrocycle containing two allyl groups was synthesized and photophysically studied. Studies were carried out on metal ion recognition and selectivity-influencing effects including the determination of the relevant thermodynamic constants as log*K* and p*K*_a_. The proposed sensor molecule is recommended for the development of Zn^2+^-selective optochemical analyzers based on covalently immobilized ionophores as it has a unique pH-independent metal ion recognition ability, which is not influenced by anions and other potentially occurring metal ions in biological samples.

## Introduction

Acridine and its derivatives have long been used as fluorophores. A search in the Web of Science® database (setting keyword acridine* results in more than 2400 hits in the titles and abstracts of publications from the last 5 years) confirms that acridines are gaining increasing attention mostly in the fields of analytical chemistry and fluorescence spectroscopy. The interest is still unbroken nowadays, as numerous new sensor molecules containing an acridine unit and their applications are reported recently [1–5]. Most commonly, a 4,5-dimethyleneacridino- or a 9-methyleneacridino-fluorophore is prepared from the commercially available acridine and then coupled to supramolecular receptor units in different ways [6–12]. Thus, signaling is induced by an indirect electron transfer mechanism. However, direct type acridino-sensor molecules containing a fluorophore as a part of the receptor unit have also been developed [13–16]. These acridino-crown ethers are most commonly used as organic- and heavy metal cation sensors [13–16]. In the case of environmental analyzers it is sufficient to physically immobilize the ionophores. On the other hand, covalent incorporation in membranes or various carrier phases is essential for biological samples as in these cases perturbation-free analysis is needed [17]. Covalent attachment generally requires additional post-synthetic modifications of complex-structured ionophores prepared by multi-step syntheses [18]. From this point of view, design of simple, easy-to-prepare and covalently immobilizable sensor molecules are preferred.

Herein, we report a new directly polymerizable bis(acridino)-macrocycle containing two allyl groups as a promising fluorescent sensor molecule for optochemical analysis of metal ions, especially Zn^2+^ in biological samples. Preliminary studies on molecular recognition and possible limitations of practical application provide a valuable starting point for future development of this type polymer-based sensors.

## Results and Discussion

### Design and Synthesis of the New Fluorescent Sensor Molecule

**Scheme 1:**
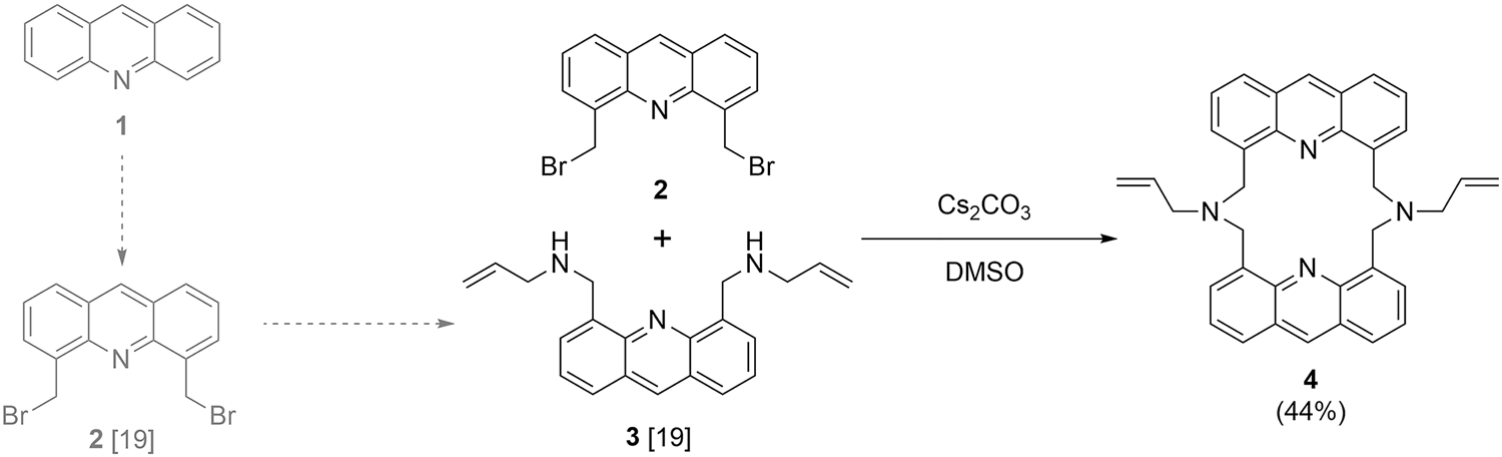
Macrocyclization of heterocyclic intermediates to gain new polymerizable fluoroionophore **4**

A directly polymerizable bis(allylamino)-ionophore containing acridine fluorophore units as parts of the coordination sphere was designed for covalent immobilization. The four nitrogens as nucleophile centers of the macrocycle are responsible for coordinating the cationic guests during molecular recognition. Moreover, the aromatic units can act as *π*-bound-donors, while the 16-crown-4 type macrocyclic cavity has an internal size comparable with several softly electrophilic metal ions. The proposed host was obtained by a simple 3-step synthetic procedure starting from acridine (**1**) involving no chromatographic purification steps. The intermediates (**2** and **3**) for macrocyclization were prepared according to the reported method [19] and were reacted in high-dilution-conditions as outlined in Scheme 1.

The reported synthetic procedure is favorable due to its simplicity and outstanding yield regarding the preparations of macrocyclic sensors. Detailed procedure for preparation and data of characterization are reported in Subsection Synthesis of 7,23-bis(prop-2-en-1-yl)-7,15,23,31-tetraazaheptacyclo[27.3.1.1^13,17^0.0^5,32^.0^9,14^.0^16,21^.0^25,30^]tetratriaconta-1,3,5(32),9,11,13(34),14,16(21),17,19,25,27,29(33),30-tetradecaene (**4**).

## Spectral Properties of the New Fluoroionophore

**Fig. 1:**
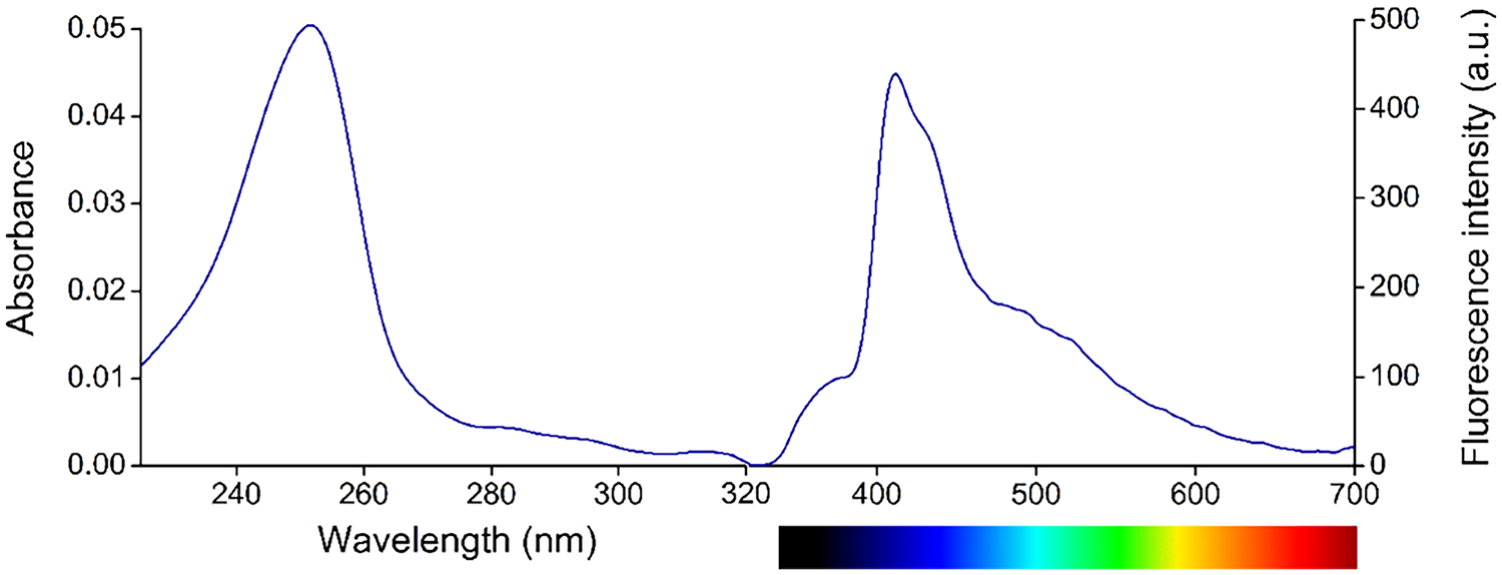
UV/Vis-absorption (left) and fluorescence emission (right, λ_excitation_ = 249 nm) spectra of sensor molecule **4**

Initially, the spectral properties of new tetraaza-macrocycle **4** were investigated. The absorption and fluorescence emission spectra of the sensor molecule are shown in Fig. 1.

The absorption and emission peak-wavelengths were 251 nm and 412 nm, respectively. A large *Stokes*-shift of 161 nm was observed. The absence of spectral overlap reduces the possibility of self-absorption. The fluorescence quantum yield was determined as 8.6 × 10^–4^ in acetonitrile, indicating a weak fluorescence of the free ligand.

## Studies on Metal Ion Selectivity and Complexation

**Fig. 2:**
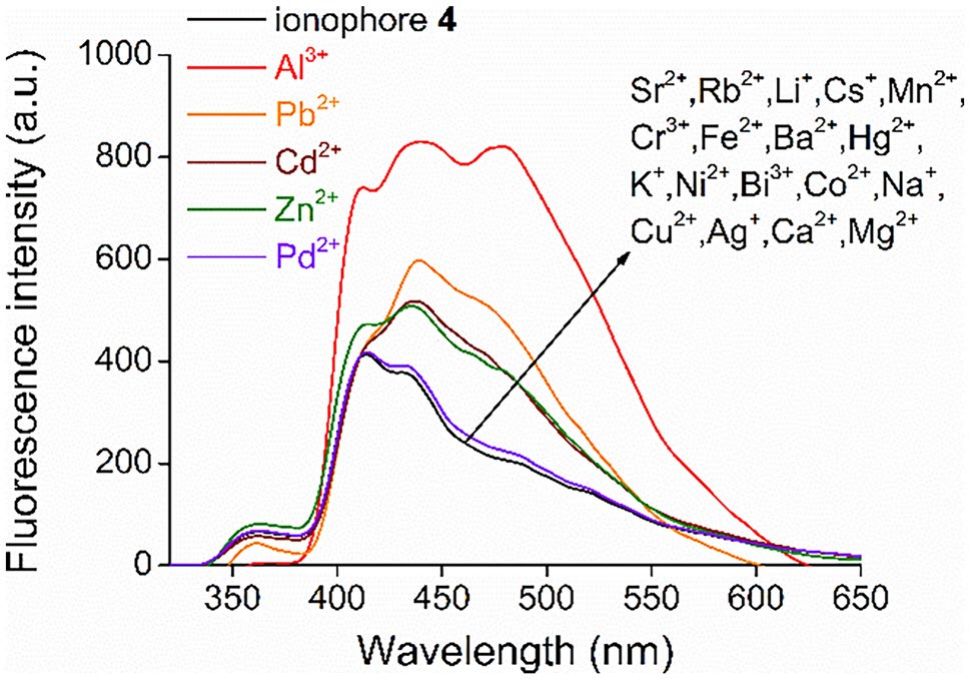
Studies on metal ion-selectivity of new fluoroionophore **4** (λ_excitation_ = 249 nm, c_host_ = 1 μM in acetonitrile, c_metal ions_ = 10 μM in water or ethanol in the case of Pd^2+^)

Studies on metal ion selectivity were performed by adding 10 equivalents of 23 different metal ions as 50 mM aqueous solutions separately to the solution of macrocycle **4** in acetonitrile (Fig. 2).

Changes in fluorescence spectra indicated complex formation with Al^3+^, Pb^2+^, Cd^2+^, Zn^2+^ and Pd^2+^. These metal ions caused a remarkable fluorescence enhancement in the corresponding order. In the cases of the other 18 metal salts, no spectral change was observed, indicating that complexation did not take place.

**Fig. 3:**
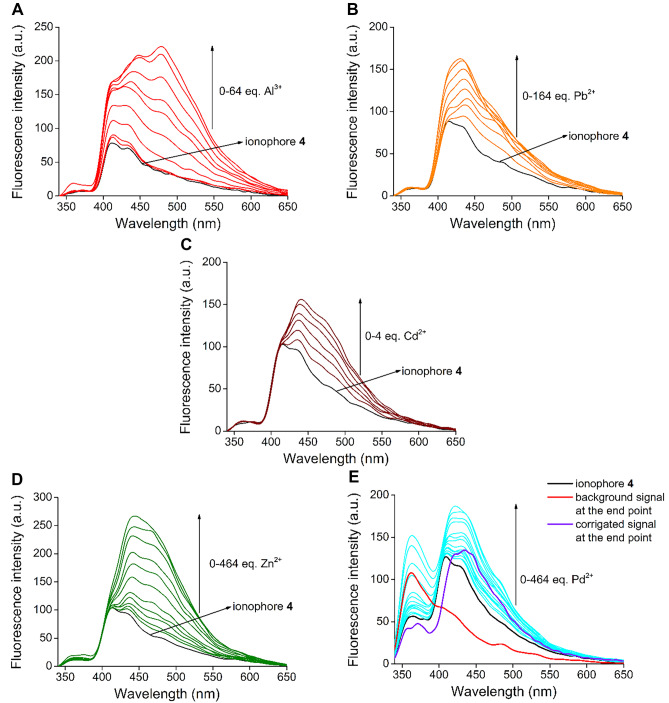
Series of spectra for fluorescence titration of new ionophore **4** with aqueous solutions of **A**: Al^3+^, **B**: Pb^2+^, **C**: Cd^2+^, **D**: Zn^2+^ and ethanol solution of **E**: Pd^2+^ (c_host_ = 1 μM in acetonitrile)

In order to determine the stability constants of the complexes, the host molecule was titrated with the aqueous solutions of the 5 preferred metal ions. Results are shown in Fig. 3.

In the case of titration with Pd^2+^, a remarkable background emission was observed, which necessitated the correction of the results over the entire spectral range. Presumably it was caused by ethanol used as a solvent, which altered the polarity of the medium during the titration experiment (PdCl_2_ as a Pd^2+^ source is insoluble in water).

**Fig. 4:**
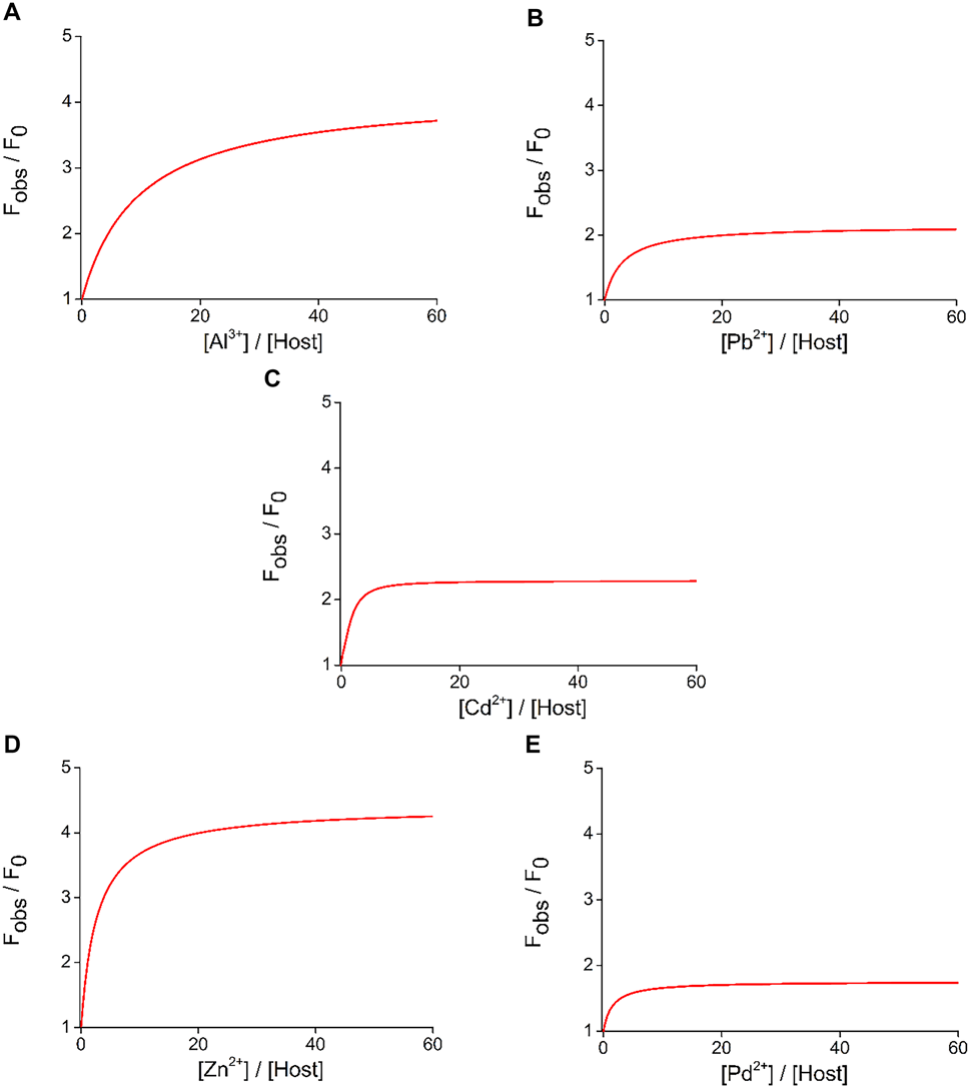
Non-linear functions as results of the applied global fitting analyses (based on Eq. 3 in Subsection Fluorescence measurements and evaluation of the results) for calculating log*K* values in the cases of the preferred ions of new fluorescent macrocycle **4** (F_obs_ refers to the observed fluorescence signal upon addition of the corresponding amount of metal ion, while F_0_ is the initial fluorescence of free host **4** in absence of metal ions)

For determining the complex stability constants, non-linear regression curves were globally fitted on the spectroscopic experimental data based on the least square’s method. The results of these regression analyses on titration experiments are shown in Fig. 4. Based on the calculations﻿, 1:1 complex stoichiometry was suggested in each case. Results are summarized in Table 1.

**Table 1:** Logarithms of the calculated *K* constants for complexes of new ionophore **4** with preferred metal ions

Metal ion	log*K*
Al^3+^	5.00 ± 0.10
Pb^2+^	5.50 ± 0.10
Cd^2+^	5.60 ± 0.10
Zn^2+^	5.70 ± 0.05
Pd^2+^	3.60 ± 0.20

Among the preferred metal ions, the most stable complexes were formed with Zn^2+^, Cd^2+^ and Pb^2+^, respectively. Similar stabilities are not surprising as these metal ions have quite the same chemical character. The host formed a complex of relatively weaker stability with Al^3+^ and Pd^2+^.

## Studies on Protonation

**Fig. 5:**
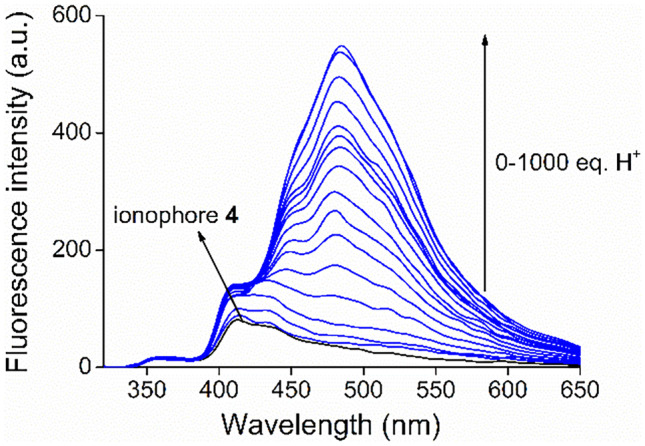
Series of fluorescence spectra for acidifying new ionophore **4** (c_ionophore_ = 1 μM in acetonitrile, λ_excitation_ = 249 nm)

New ionophore **4** is prone to accept protons in acidic medium due to its weak basic character. Studies on protonation are essential, since it can strongly influence optical signaling. Moreover, the different ionization states also effect the metal ion recognition ability of the sensor molecule. For characterizing the proton association ability of the new macrocycle, its p*K*_a_ was determined. Determination was carried out in acetonitrile due to the poor water-solubility of the ionophore. Nitric acid was gradually added to the solution of macrocycle **4**. The aliphatic nitrogen atoms of the ionophore were protonated immediately, while protonation of the heteroaromatic ones took place gradually (Fig. 5). The bathochromic shift of the emission indicated the appearance of a new molecular form.

**Fig. 6:**
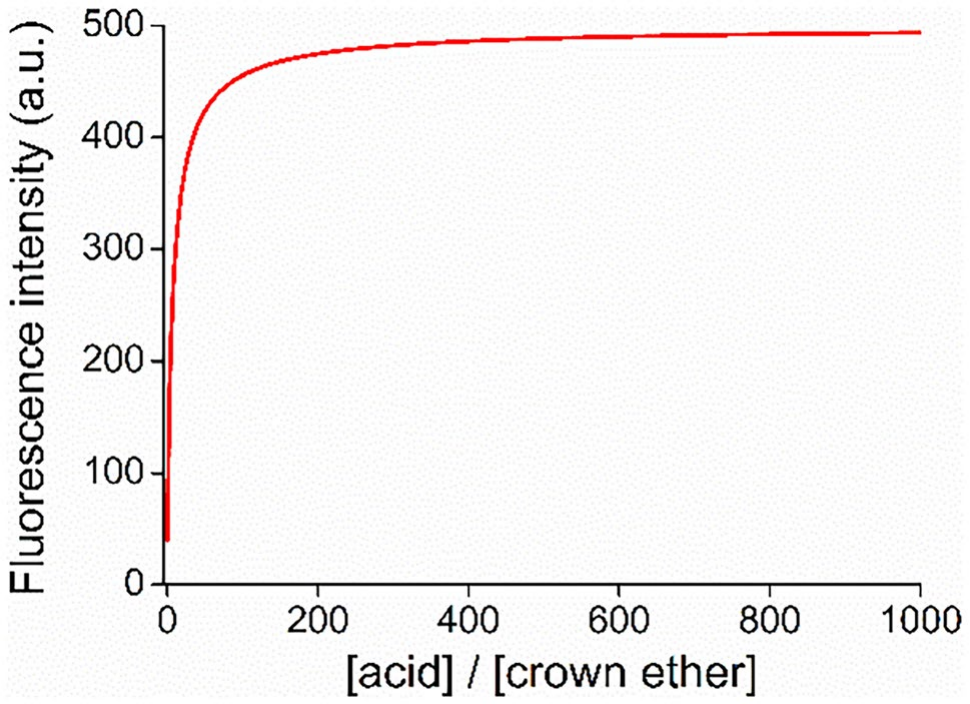
Non-linear regression curve to determine the p*K*_a_ (based on Eq. 6 in Fluorescence measurements and evaluation of the results).

Determination of p*K*_a_ was also carried out according to a non-linear global fitting using the least square’s method (Fig. 6). The p*K*_a_, which characterizes the deprotonation of the heteroaromatic *NH*^+^ unit of the ionophore was found as 9.73 ± 0.03 in acetonitrile. Studies showed, that the protonation of only one heterocyclic unit took place and the protonation of the second acridine unit began much later. It suggests the formation of intramolecular stabilizing interactions.

## Studies on Anion-Coordination

**Fig. 7:**
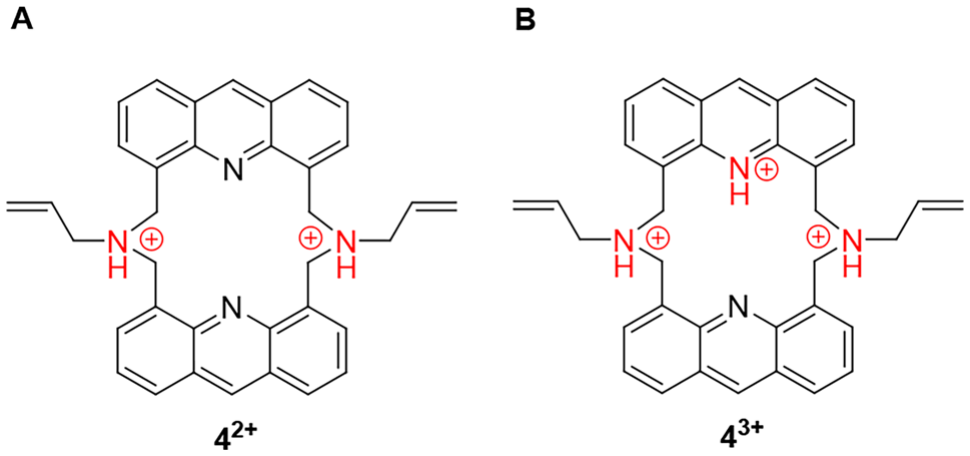
The protonated forms of ionophore **4 A**: in a neutral water-acetonitrile mixture (**4**^**2+**^) and **B**: in an acidic water-acetonitrile mixture at the end point of acid titration (**4**^**3+**^)

The p*K*_a_ of the conjugate acid of the aliphatic tertiary amine groups of the ionophore are above 8 (predicted by ChemAxon), which indicates that these aliphatic *N*-atoms are mainly in their protonated forms in neutral aqueous medium. Hence, studies on anion-coordination were carried out to exclude the possibility of interference with several commonly occurring counterions. Furthermore, investigations were also carried out on the anion-coordinating ability of the triple-protonated macrocycle as its molecular recognition ability can strongly differ from that of the corresponding double-positively charged or the neutral one. The mentioned ionization forms are shown in Fig. 7.

**Fig. 8:**
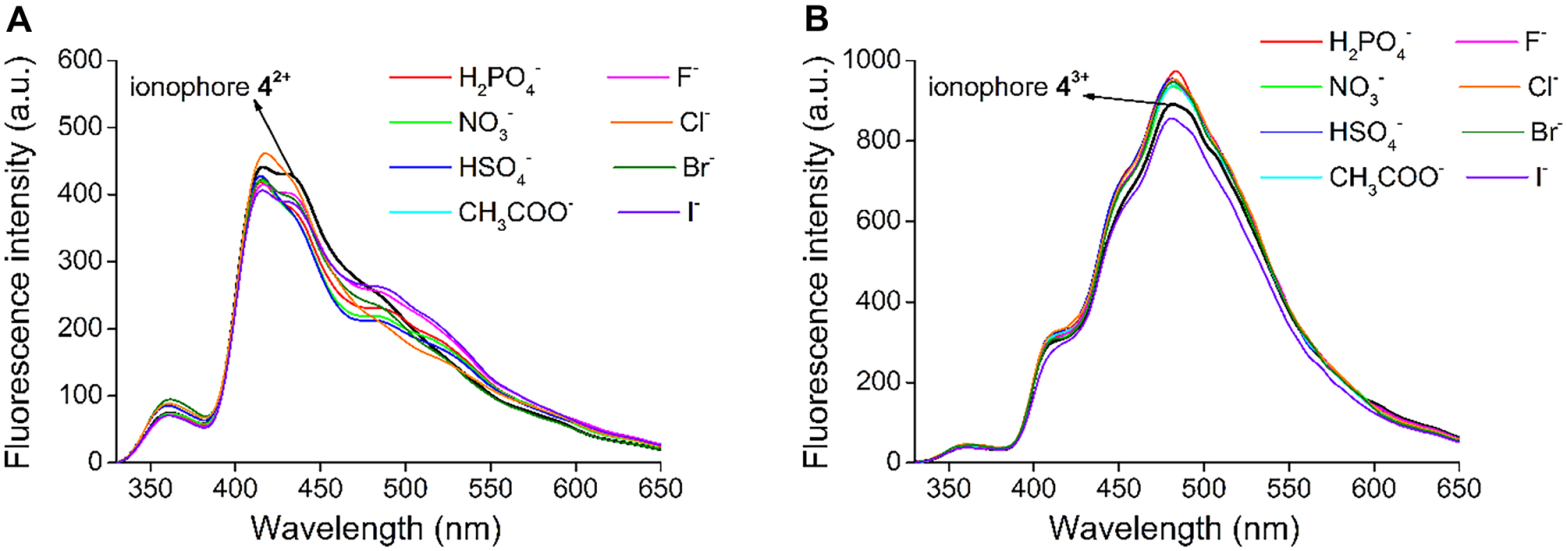
Studies on the anion-selectivity of double-protonated **4**^**2+**^ (**A**) and triple-protonated **4**^**3+**^ (**B**) of ionophore **4** (c_ionophore_ = 1 μM, λ_excitation_ = 249 nm) in an acetonitrile-based semi-aqueous medium

Various tetrabutylammonium (sterically shielded cation which cannot be complexed) salts of H_2_PO_4_^−^, NO_3_^−^, HSO_4_^−^, CH_3_COO^−^, F^−^, Cl^−^, Br^−^ and I^−^ in 50 mM aqueous solutions were added to differently protonated ionophore **4** in an amount of 10 equivalents regarding to the host (Fig. 8). No significant spectral change was observed in each case, indicating that complexation of anions did not take place even in the protonated forms of the host molecule.

## Conclusions

We have designed and synthesized a new fluorescent bis(acridino)-macrocycle containing two allyl groups as an optochemical sensor molecule. This host molecule was obtained with a good yield by a simple three-step synthetic procedure starting from acridine. Photophysical studies showed a favorable large *Stokes*-shift. New ionophore **4** has negligible fluorescence as a free ligand and gave a turn-on optical response by adding Al^3+^, Pb^2+^, Cd^2+^, Zn^2+^ and Pd^2+^ among 23 metal ions. Studies on metal ion recognition established the formation of stable complexes in the cases of Al^3+^, Pb^2+^, Cd^2+^ and Zn^2+^ with a 1:1 stoichiometry. These preferred metal ions can selectively be detected over a wide pH-range as the different protonated states of the sensor molecule had no significant effect on complexation. The presence of various anions did also not influence the metal ion recognition. From the aspect of practical application, the proposed sensor molecule can effectively be used in biological samples for analysis of Zn^2+^ since in this case the occurrence of competing Al^3+^, Pb^2+^ and Cd^2+^ is not expected. The host molecule contains two allyl groups, which make it suitable for direct polymerization or covalent immobilization. This structural property enables various future applications.

## Experimental

### Chemicals and Apparatus

Chemicals were purchased from Sigma-Aldrich Corporation (USA, owned by Merck, Germany) and used without further purification unless otherwise noted. Solvents were dried and purified according to well established methods [20]. Aluminum oxide 60 F254 neutral type E (Merck, Germany) plate was used for thin-layer chromatography (TLC). Melting point was taken on a Boetius micro-melting point apparatus and is uncorrected. Infrared spectrum was recorded on a Bruker Alpha-T FT-IR spectrometer (Bruker Corporation, USA) using KBr pastilles. NMR spectra were recorded on a Bruker 300 Avance spectrometer (Bruker Corporation, USA; at 300 MHz for ^1^H and at 75.5 MHz for ^13^C spectra). HRMS analysis was performed on a Thermo Velos Pro Orbitrap Elite (Thermo Fisher Scientific, Germany) system. The ionization method was ESI and operated in positive ion mode. The protonated molecular ion peaks were fragmented by CID at a normalized collision energy of 35–45%. Data acquisition and analysis were accomplished with Xcalibur software version 2.2 (Thermo Fisher Scientific, Germany).

UV/Vis spectra were recorded on a UNICAM UV4-100 spectrophotometer controlled by VIZION 3.4 software (ATI UNICAM, UK). Fluorescence emission spectra were recorded on a Perkin-Elmer LS 50B luminescent spectrometer (PerkinElmer Inc., USA) and were corrected by FL Winlab 3.0 spectrometer software (PerkinElmer Inc., USA). Quartz cuvettes with path length of 1 cm were used in all cases.

### Synthesis of 7,23-bis(prop-2-en-1-yl)-7,15,23,31-tetraazaheptacyclo[27.3.1.1^13,17^0.0^5,32^.0^9,14^.0^16,21^.0^25,30^]tetratriaconta-1,3,5(32),9,11,13(34),14,16(21),17,19,25,27,29(33),30-tetradecaene (4)

A mixture of secondary amine **3** [19] (416 mg, 1.32 mmol), finely powdered anhydrous caesium carbonate (2136 mg, 6.56 mmol) and dry and pure DMSO (200 mL) were stirred vigorously under argon atmosphere at room temperature for 30 min, then bromo-derivative **2** [19] (480 mg, 1.32 mmol) in dry and pure DMSO (50 mL) was added dropwise. The temperature of the mixture was raised to 60 °C and kept at this temperature for 4 days. Water (500 mL) was added to the reaction mixture, which was extracted with ethyl acetate (4 × 500 mL). The combined organic phase was shaken with water (9 × 500 mL) and then with saturated aqueous sodium chloride solution (1 × 500 mL) to remove DMSO. The organic phase was dried over magnesium sulphate, filtered and the solvent was evaporated under reduced pressure. The crude product was recrystallized from methanol, then from propane-2-ol to gain **4** (304 mg, 44%) as dark yellow crystals.

M.p. = 144 °C. *R*_f_ = 0.50 (Al_2_O_3_ TLC, propane-2-ol:dioxane 1:5). ^1^H-NMR (CD_3_OD): δ [ppm]: 3.67 (d, *J* = 6.1 Hz, 4H); 4.99 (s, 8H); 5.33 (d, *J* = 10.4 Hz, 2H); 5.55 (d, *J* = 17.3 Hz, 2H); 6.18–6.26 (m, 2H); 7.32 (t, *J* = 7.6 Hz, 4H); 7.67–7.71 (m, 8H); 8.54 (s, 2H). ^13^C-NMR (CD_3_OD): δ [ppm]: 53.93; 56.19; 116.77; 124.34; 127.23; 129.79; 132.08; 135.89; 144.29; 154.75. IR: ν_max_ [cm^−1^]: 3040; 2956; 2924; 2853; 1676; 1640; 1617; 1534; 1452; 1261; 1171; 1106; 1021; 916; 759. HRMS: m/z = [MH^+^]: 521.2699, (Calcd. for C_36_H_32_N_4_, 520.2627).

## Fluorescence Measurements and Evaluation of the Results

Spectroscopic measurements were carried out at room temperature (25 ± 1 °C). Polarizers were not applied. A 350 nm cut off type bandpass filter and 5 nm excitation and emission slits were used in the cases of titration experiments, while in the other cases slits were 10 nm. During spectrophotometric titrations, the solutions were added with a *Hamilton*-syringe to the acetonitrile solutions of the ligand. The results were corrected with the background signal and the dilution effect of the added solutions. OriginPro 8.6 (OriginLab Corp., USA) software was used for evaluation and visualization of the spectroscopic results.

Relative quantum efficiencies were also determined in acetonitrile according to a literature method [21] based on a comparison with acridine as a standard [22]. The excitation and emission spectra were recorded in the same conditions and instrument settings as in the case of the standard. (The excitation wavelength for the ionophore was chosen to be 249 nm, because of better comparability with the fluorophore subunit and the reference compound.) The following equation was used for calculation:

1 $$\frac{{\Phi }_{i}}{{\Phi }_{r}}=\frac{{n}_{i}^{2}}{{n}_{r}^{2}}\bullet \frac{{\int }_{0}^{\infty }{I}_{i}\left({\lambda }_{ex},{\lambda }_{em}\right)d{\lambda }_{em}}{{\int }_{0}^{\infty }{I}_{r}\left({\lambda }_{ex}, {\lambda }_{em}\right)d{\lambda }_{em}}\bullet \frac{1-{10}^{{-A}_{r}\left({\lambda }_{ex}\right)}}{1-{10}^{-{A}_{i}\left({\lambda }_{ex}\right)}}$$

where subscript *i* refers to the sample of the initial investigated compound, while subscript *r* refers to the reference. The $\Phi$ is the quantum yield, *n* is the respective refractive index of the solvents, *I* is the fluorescence intensity, ${\lambda }_{ex}$ is the excitation wavelength, ${\lambda }_{em}$ is the emission wavelength and *A* is the absorbance.

In the cases of turn-on type optical response, the stability constants of the complexes (*K*) were determined by global non-linear regression analysis. For determination of the complex stability constant based on the observed fluorescence enhancement upon complexation, the following equation was used [23]:

2 $$F={I}_{0}\Phi \varepsilon b\left[\mathrm{X}\right]={k}_{X}\left[\mathrm{X}\right]$$

where *F* is the measured fluorescence intensity, *I*_*0*_ is the intensity of the emission, Φ is the fluorescence quantum yield, *ε* is the molar absorption coefficient, *b* is the optical path length, [X] is the molar concentration of species X and *k*_*X*_ is a constant referring to the optical properties of species X.

In the case of a complex with 1:1 stoichiometry the association constant can be calculated by the following equation:

3 $$\frac{\text{F}}{{\text{F}}_{0}}{=}\frac{{{\text{k}}_{\text{H}}}/{{\text{k}}_{\text{H}}^{0}}{+}\left({{\text{k}}_{\text{HG}}}/{{\text{k}}_{\text{H}}^{0}}\right){\text{K}}_{\text{a}}[\mathrm{G}]}{\text{1} + {\text{K}}_{\text{a}}\text{[G]}}$$

where the ratios of *k* parameters and *K*_*a*_ were left as floating parameters during the fitting method. (In the absence of *K*_*a*_, *k* values also have to be initially set as floating parameters. For determination of *K*_*a*_ values, ratios of fitted *k* values were handled as constants.) Parameters *F* and *F*_*0*_ are wavelength-dependent variables and [G] was set as a variable, too. *F*_*0*_ refers to the initial fluorescence intensity of the free host molecule, *k*_*H*_ is a constant referring to the optical properties of the free host molecule, ${k}_{H}^{0}$ is a constant referring to the optical properties of the free host in the presence of the preferred guest molecule, constant *k*_*HG*_ describes the photophysical features of the complex, *K*_*a*_ is the association constant and [G] is the concentration of the initial guest molecules.

Global non-linear fitting was carried out similarly in the case of complexes with 1:2 (host:guest) stoichiometry based on the following equation:

4 $$\Delta {F}_{obs}=\frac{{k}_{\Delta HG}{\left[\mathrm{H}\right]}_{0}{K}_{1}\left[\mathrm{G}\right]+{k}_{\Delta {HG}_{2}}{\left[\mathrm{H}\right]}_{0}{K}_{1}{K}_{2}{[\mathrm{G}]}^{2}}{1+{K}_{1}\left[\mathrm{G}\right]+{K}_{1}{K}_{2}{[\mathrm{G}]}^{2}}$$

where *ΔF*_*obs*_ is the change in fluorescence during titration steps, *k*_*ΔHG*_ = *k*_*HG*_—*k*_*H*_, [H]_0_ is the initial concentration of the host, *K*_*1*_ is the association constant of the first step of the complex formation equilibrium, while *K*_*2*_ is the association constant of the second step of the complexation.

The described method for the complexes with a 2:1 (host:guest) stoichiometry was performed based on the following mathematical formula:

5 $$\Delta {F}_{obs}=\frac{{k}_{\Delta HG}{\left[\mathrm{G}\right]}_{0}{K}_{1}\left[\mathrm{H}\right]+{k}_{{\Delta HG}_{2}}{\left[\mathrm{G}\right]}_{0}{K}_{1}{K}_{2}{\left[\mathrm{H}\right]}^{2}}{1+{K}_{1}\left[\mathrm{H}\right]+{K}_{1}{K}_{2}{[\mathrm{H}]}^{2}}$$

where [G]_0_ is the initial concentration of the guest molecule and [H] is the concentration of the free host molecules.

The studies of the complex stoichiometries were also carried out applying the described global non-linear fitting methods. The stoichiometries were determined based on the deviations resulting from the parameter fitting according to the least square’s method using the mentioned equations. Titration experiments were performed with careful consideration of the relevant recommendations [24].

Determination of p*K*_a_ values in non-aqueous medium was carried out based on the following equation [25]:

6 $$F= \frac{{F}_{max}{[{\mathrm{H}}^{+}]}^{n}+{F}_{min}{K}_{acid}}{{K}_{acid}+{[{\mathrm{H}}^{+}]}^{n}}$$

where *F* is the measured fluorescence intensity, *F*_*max*_ is the fluorescence intensity at the starting point of acid titration, [H^+^] refers to the proton concentration, *n* shows the number of associated proton / molecules, *F*_*min*_ is the fluorescence intensity at the end point of acid titration, *K*_*acid*_ is the acid dissociation constant of the investigated compound. During the fitting method, the *n* and the *K*_*acid*_ were defined as floating parameters in the equation. The value of *n* proved to be close to 1, thus it was set as a constant. Hence, based on the known values of variable [H^+^] and the wavelength-dependent variables *F*, *F*_*max*_, *F*_*min*_, parameter *K*_*acid*_ can be determined.

During the calculation it was considered that the proton dissociation of nitric acid is strongly reduced in acetonitrile compared to the estimated total dissociation of protons in water. The p*K*_a_ for nitric acid in acetonitrile is 10.6 [26]. The [H^+^] values in Eq. 6 were corrected with the degree of dissociation corresponding to the concentration of nitric acid using the *Ostwald*’s dilution law.

## Data Availability

The datasets generated during and/or analysed during the current study are available from the corresponding author on reasonable request.
